# Cause-specific mortality patterns among hospital deaths in Thailand: validating routine death certification

**DOI:** 10.1186/1478-7954-8-12

**Published:** 2010-05-18

**Authors:** Junya Pattaraarchachai, Chalapati Rao, Warangkana Polprasert, Yawarat Porapakkham, Wansa Pao-in, Noppcha Singwerathum, Alan D Lopez

**Affiliations:** 1 Department of Community Medicine, Thammasat University, Pathumthani, Thailand; 2 School of Population Health, University of Queensland, Brisbane, Australia; 3 School of Health Sciences, Sukhothaithummathirat Open University, Bangkok, Thailand; 4 Ministry of Public Health, Bangkok, Thailand; 5 Department of Surgery, Thammasat University, Pathumthani, Thailand; 6 Health Information Systems Knowledge Hub, University of Queensland, Brisbane, Australia

## Abstract

**Background:**

In Thailand, 35% of all deaths occur in hospitals, and the cause of death is medically certified by attending physicians. About 15% of hospital deaths are registered with nonspecific diagnoses, despite the potential for greater accuracy using information available from medical records. Further, issues arising from transcription of diagnoses from Thai to English at registration create uncertainty about the accuracy of registration data even for specified causes of death. This paper reports findings from a study to measure validity of registered diagnoses in a sample of deaths that occurred in hospitals in Thailand during 2005.

**Methods:**

A sample of 4,644 hospital deaths was selected, and for each case, medical records were reviewed. A process of medical record abstraction, expert physician review, and independent adjudication for the selection and coding of underlying causes of death was used to derive reference diagnoses. Validation characteristics were computed for leading causes of hospital deaths from registration data, and misclassification patterns were identified for registration diagnoses. Study findings were used to estimate cause-specific mortality patterns for hospital deaths in Thailand.

**Results:**

Adequate medical records were available for 3,316 deaths in the study sample. Losses to follow up were nondifferential by age, sex, and cause. Medical records review identified specific underlying causes for the majority of deaths that were originally assigned ill-defined causes as well as for those originally assigned to residual categories for specific cause groups. In comparison with registration data for the sample, we found an increase in the relative proportion of deaths in hospitals due to stroke, ischemic heart disease, transport accidents, HIV/AIDS, diabetes, liver diseases, and chronic obstructive pulmonary disease.

**Conclusions:**

Registration data on causes for deaths occurring in hospitals require periodic validation prior to their use for epidemiological research or public health policy. Procedures for death certification and coding of underlying causes of death need to be streamlined to improve reliability of registration data. Estimates of cause-specific mortality from this research will inform burden of disease estimation and guide interventions to reduce avoidable mortality in hospitals in Thailand.

## Introduction

The optimal source of information on causes of death in populations is vital registration data based on medical certificates of cause of death, issued by attending physicians. The World Health Organization (WHO) has prescribed a standard form for the medical certificate of cause of death, which allows for the listing of multiple diseases or conditions that occur in a chronological and pathophysiological sequence terminating in death, as well as the mention of associated diseases or conditions that are not directly linked to the causal sequence [1]. In each case, the listed causes are usually based on the attending physician's firsthand knowledge of the illness and circumstances leading to death or on information derived from available medical records that support any observations during the terminal phase. In some instances, deaths in hospitals are also subjected to pathological autopsies to determine the cause, but these are becoming increasingly rare, reserved largely for medico-legal cases [2].

Medical records for individuals admitted to hospitals in the days prior to death usually contain sufficient clinical, laboratory, and imaging evidence to support clinical diagnoses and guide therapeutic measures. On the other hand, medical records may be relatively uninformative for cases in which death occurs soon after admission, unless there is adequate linkage and availability of records from previous contact with health services. Doctors on emergency duty outside routine working hours are sometimes required to attend to dying patients and to subsequently certify the cause of death, and will often have to rely on available medical records for clinical inputs into death certification. Hence, the adequacy of medical records in hospitals has an important bearing on the accuracy of causes of death entered on the death registration certificate. Research conducted in several countries has indicated that even where adequate medical records are available, causes of death filed at registration are not always reliable. Such discrepancies can have important implications for the use of these data in epidemiological research and the determination of public health priorities [3,5].

In Thailand, about 35% of all deaths occur in hospitals. Each hospital death is medically certified as to cause by attending physicians, using a format that conforms to the international standard prescribed by WHO. Previous research has indicated considerable problems with the quality of medical records in hospitals in Thailand [6,8] as well as with the process of transcribing diagnoses recorded in English on death certificates into Thai for registration purposes [9]. As a result, about 15% of hospital deaths in Thailand are registered with nonspecific diagnoses. This is unacceptably high, given the potential for detailed clinical information to better support accurate diagnoses of these deaths, and was identified as one of the priority areas for improvement in the Thai vital registration system [10]. Furthermore, the problems arising from transcription create additional uncertainty about the accuracy of specified causes for hospital deaths [8], limiting the utility of registration data on causes of death.

This paper reports the methods and findings from research conducted to investigate the validity of registered causes in a sample of hospital deaths in Thailand during 2005. The sample included deaths with specified as well as nonspecified causes, including deaths coded to vague categories such as ill-defined heart disease that have no utility for epidemiological analyses or public health policy. The overall goal of the study was to utilize the research findings to develop a best estimate of cause-specific mortality for the almost 150,000 deaths that occur in hospitals in Thailand each year, under the assumption that the issues governing the validity of causes in the study sample are similar to those for all deaths in health facilities in Thailand. We have also sought to assess factors that influence the validity of registration diagnoses in the study sample. These include assessments of the quality of evidence in medical records, the quality of cause-of-death certification practices, and the application of rules for selection and coding of underlying causes of death, according to the 10th revision of the International Classification of Diseases and Health Related Problems (ICD-10). An associated goal of the study was to strengthen technical capacity in Thailand for medical records evaluation, cause-of-death certification and coding, and mortality data analysis. The findings from the research about prevailing cause-specific mortality patterns, as well as the capacity-building measures, have important implications for current and future health priorities in Thailand and the monitoring and evaluation of health programs.

## Methods

### Study design and sampling plan

The overall design and sampling methodology for the study has been described elsewhere[11]. In brief, the inclusion criteria for the primary sampling unit (death) was that the deceased should have been a registered Thai citizen and a permanent resident in the province selected into the study. A nationally representative study sample of 11,984 deaths was selected from the national death registration database for 2005 using a stratified cluster sampling approach. This sample was distributed across 28 districts in nine provinces in Thailand. In this paper, we report findings from investigation of 4,644 deaths in the study sample that had occurred in hospitals, of which about 90% occurred in government health facilities at the district, provincial, and metropolitan levels, with the remaining 10% from private hospitals, mostly in Bangkok.

### Data collection and processing

For each sampled death, a household visit was conducted to confirm the event. If the death had occurred in a hospital, consent was obtained to access relevant medical records. Available hospital medical records were first de-identified to maintain confidentiality and subjected to detailed review as follows. In each province, a team of three physician reviewers was established, comprising specialists from internal medicine, surgery, pediatrics, or gynecology. Reviewers received specific training in cause-of-death certification as well as in the selection and coding of the underlying cause of death according to ICD-10 principles. Training programs were followed by test clinical scenarios, with 10% reliability checks to assure consistency in certification and coding practice across reviewers.

For each record, reviewers first filled in a detailed abstraction form, based on models used elsewhere [5,12]. The abstraction form included clinical details from the medical history and physical examination, laboratory and imaging studies, treatment details, and clinical course until death. Subsequently, reviewers completed a standard international form of medical certificate of cause of death for each case. All completed death certificates were then processed by teams of three trained coders in each province who assigned relevant ICD codes for each cause of death listed on the certificate. Each coded certificate was then returned to the concerned physician reviewer, who applied ICD-10 rules to select the underlying cause of death. Where necessary, a second opinion from a colleague within the team was sought to achieve consensus on the underlying cause of death. Reviewers also assigned a qualitative label as to whether the medical record contained confirmatory or weak evidence to support the diagnosis of the underlying cause of death. Available histopathology, laboratory, or imaging evidence was deemed confirmatory evidence for individual causes, while findings from only clinical history and physical examination were considered weak evidence.

All abstracted records along with coded death certificates were submitted to a central expert team comprising three specialists from internal medicine with vast experience in cause-of-death certification and ICD coding. Members of the team reviewed the material for each case and revised the selection of, or code for, the underlying cause of death as required. For descriptive analysis, revisions were characterized as "disagreement" or "incorrect code," depending on whether the revised code resulted in a change in category under the ICD Mortality Tabulation List 1 [13] or not.

In four provinces (Chiang Rai, Ubolrajthani, Supanburi, and Songkhla), additional research was conducted to audit the quality of information on death certificates filed at registration. A total of 2,232 cases were included in this component, and the audit was independently conducted by the medical record review team for Bangkok. For each case, a detailed scoring system was used to assess the accuracy, level of detail, and consistency in the sequence of listed causes in the registration death certificate compared to the reference cause-of-death certificate derived from medical record review. The audit also reviewed the general quality of information in the medical records, including use of abbreviations, ambiguous terminology, and legibility.

Using the medical records review diagnoses as reference standards, validation characteristics were computed for the routine registration data in terms of concordance, sensitivity, and positive predictive value for leading causes of death. Systematic patterns of misclassification by cause were identified. Finally, the study findings were used to estimate the corrected proportionate mortality distributions by cause and sex across three age groups: 15-49 years; 50-69 years; and 70 years and over, for which adequate deaths were available in the sample.

## Results

Table 1 shows the age distribution, by sex, of all hospital deaths in Thailand reported in the registration data, and for cases recruited into the study. The age groups for which data are presented correspond broadly to ages when cause-specific mortality is likely to change. Overall, the field sample does not indicate any major distortions by sex or across age groups and can be considered a satisfactory representation of hospital deaths in the vital registration data.

**Table 1:** Age-sex distribution of hospitals deaths (in %), according to registration data and in the study sample, Thailand, 2005.

Age Group	Males		Females	
	
	Vital Registration	Field Sample	Vital Registration	Field Sample
< 1 year	3.3	2.3	3.7	2.1
1 - 14 years	2.3	1.6	2.2	1.6
15 - 49 years	35.3	35	22.7	21.8
50 - 74 years	41.0	41.7	43.5	45
> 75 years	18.1	19.2	27.9	29.4
All ages (100%)	82601	1944*	57930	1365*

*Excluding 2 male and 5 female deaths for which age could not be verified

**Figure 1:**
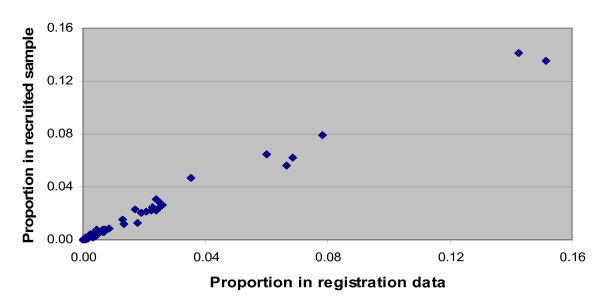
**Comparison of cause-of-death distributions for hospital deaths from registration data and registered causes in the recruited study sample, Thailand, 2005**.

Figure 1 compares the distribution of the 20 leading causes of hospital deaths in Thailand from the vital registration data (i.e., the sampling frame) to the distribution of the same causes in the sample of hospital deaths recruited into the study. Again, the two cause-of-death distributions are quite similar, suggesting that any losses to follow up were nondifferential by cause, supporting the generalizability of study findings.

Table 2 provides a population-level assessment of the validation characteristics and implications of misdiagnoses for the leading causes of hospital deaths as reported in the vital registration system. Of principal interest is the reassignment of deaths, originally classified to nonspecific categories in the registration data, to specific causes of death upon medical record review. Ill-defined causes and septicemia (which is an immediate rather than underlying cause of death) together account for almost 30% of hospital deaths in vital registration, but on reassessment, are collectively estimated to cause less than 2% of deaths. Proportionate mortality from pneumonia, also often the immediate rather than underlying cause of death, was halved upon review, from 6.2% to 3.4%. Several "other" or residual categories for major disease and injury groups also declined substantially after review, particularly "all other external causes," "other infectious diseases," and "other respiratory diseases." The reassignment of deaths from the above nonspecific categories upon medical record review resulted in a substantial increase (10% to 70%) in the relative importance of cerebrovascular disease (11.6% of all hospital deaths), ischemic heart disease (8.1%), transport accidents (6.4%), HIV/AIDS (7.7%), liver diseases (primarily cirrhosis) (4.3%), chronic obstructive pulmonary diseases (COPD) (4.8%), and lung cancer (3.3%) as leading causes of deaths in Thai hospitals (males and females combined). In addition, the medical record review assigned deaths to several important causes that have not been categorized in Table 2, and are currently listed under the "Final" column as 1,004 deaths due to "other causes." These include 260 deaths from several site-specific cancers (colorectal, stomach, breast, cervix, prostate, and others), 199 deaths from diabetes, 146 deaths from other specific external causes (falls, suicide, and assault), 112 deaths from cardiovascular conditions such as hypertensive and rheumatic heart disease, and the remaining from several other less prominent causes.

**Table 2:** Validation characteristics for hospital deaths in Thailand, 2005.

		Medical records review						
						
Cause	As in VR data	Matched	Assigned to other causes	Assigned from other causes	Final	Sensitivity (95% CI)	Positive Predictive value (95% CI)	% change in mortality proportion
Septicaemia	470	19	451	10	29	65.5 (48,82)	4.1 (2, 6)	-1517.2
Ill-defined conditions	447	15	432	14	29	51.7 (34 70)	3.4 (2, 5)	-1441.4
Cerebrovascular diseases	262	203	59	183	386	52.7 (48, 58)	77.4 (72, 82)	31.9
Ischaemic heart disease	241	138	76	129	267	51.7 (46, 58)	64.5 (58, 71)	19.9
Pneumonia	207	44	163	68	112	39.3 (30, 48)	21.3 (16. 27)	-84.8
All other external causes	185	17	168	25	42	40.5 (26, 55)	9.2 (5, 13)	-340.5
Other genitourinary diseases	156	51	105	86	137	37.2 (29, 42)	32.7 (25. 40)	-13.9
Lung cancer	102	85	19	26	111	76.6 (69, 84)	83.3 (76, 91)	8.1
Road traffic accidents	92	91	1	122	213	42.7 (36, 49)	98.9 (97, 100)	56.8
Disease of the liver	86	63	23	84	149	42.9 (35, 51)	73.3 (64, 83)	41.5
HIV/AIDS	83	79	4	177	256	30.9 (25, 36)	95.2 (91, 100)	67.6
Other malignant neoplasms	79	24	55	28	52	43.2 (23, 60)	30.4 (20, 41)	-51.9
COPD	77	54	23	105	159	34 (27, 41)	70.1 (66, 80)	51.6
Other digestive diseases	74	17	57	47	64	26.6 (16, 37)	23 (13, 33)	-15.6
Other respiratory diseases	72	10	62	13	23	43.5 (23, 68)	13.9 (6, 22)	-213.0
Other heart diseases	71	14	57	68	82	17.1 (9, 25)	19.7 (11, 29)	13.4
Liver cancer	68	58	10	27	85	68.2 (58, 78)	85.3 (77, 94)	20.0
Other infectious diseases	52	6	46	21	27	22.2 (7, 39)	11.5 (3, 20)	-92.6
Perinatal conditions	42	37	5	12	49	75.5 (63, 88)	88.1 (78, 98)	14.3
Tuberculosis	40	13	27	29	42	31 (17, 45)	32.5 (18, 47)	4.8
All other causes	437				1004			
								
**Total**	3316				3316			

Observed sensitivity scores are low for most causes of death, due to the reassignment of deaths from nonspecific categories. Positive predictive values, on the other hand, are more indicative of the validity of registration diagnoses because they show the proportion of deaths registered from a specific cause that are actually due to that cause upon medical record review. As expected, these scores are comparatively low for the nonspecific categories but are higher for specific causes such as cerebrovascular diseases, site-specific cancers, and transport accidents, among others. While these findings provide some confidence in cause-of-death certification and coding among registration data for these causes, it is important to understand in more detail the nature and extent of misclassification patterns in the registration data to guide remedial action.

Table 3 shows the principal patterns of misclassification by cause for the leading causes of registered death in hospitals. Cases of septicemia (470 deaths) were primarily reassigned to cerebrovascular diseases (55), HIV/AIDS (44), and pneumonia (38), whereas ill-defined deaths (447) tended to be true cases of ischemic heart disease (75), other heart diseases (36), COPD (39), and cerebrovascular disease (25). The misclassification patterns for deaths registered to other residual categories cover a broad range of specific causes, particularly for "other respiratory diseases" and "other heart diseases." Lung and liver cancer have relatively few cases distributed to, or from other causes, suggesting that these causes are being diagnosed reasonably accurately in hospital death statistics. Perhaps the most important change observed from the study is the gross underdiagnosis of diabetes as a registered underlying cause among hospital deaths in the sample, with the medical record review indicating about six times more deaths from diabetes in the sample. This may well have led to an underappreciation of the importance of diabetes as a public health issue in Thailand. The observation that misclassification patterns for specific and nonspecific categories are not entirely compensatory suggests that there is substantial uncertainty in the registration data for specific, important causes of death, limiting their value for any appreciation of population-wide cause-specific mortality patterns in Thailand.

**Table 3:** Misclassification patterns for leading causes of hospital deaths in the study.

Causes of death *	Medical records diagnoses																
**Vital registration diagnoses**	**20**	**31**	**34**	**46**	**52**	**66^†^**	**67**	**68**	**69**	**74**	**76**	**80**	**81**	**84**	**96**	**All other causes**	**Total**

Septicaemia (12)	44	2	3	3	53	6	8	3	55	38	16	27	19	47	2	144	**470**
Ill defined conditions (94)	16	6	7	5	27	16	75	36	25	14	39	10	14	13	9	135	**447**
Cerebrovascular diseases (69)			1		7	1	4	5	203					1	9	31	**262**
Ischaemic heart diseases (67)	1		2		26	5	138	9	3	2	3		3	6		16	**214**
Pneumonia (74)	40		3		9	1	4	2	25	44	21	7	1	10	3	37	**207**
All other external causes (103)					1	1	2	1	25	1					93	61	**185**
Genitourinary diseases (84)	1	1		1	37	24	2	3	3	1	1	5	2	58		17	**156**
Lung cancer (34)		1	85	6					1		4					5	**102**
Transport accidents (96)								1							91		**92**
Liver diseases (80)	2	2			1		2		2			63	2	1		11	**86**
HIV/AIDS (20)	79											1				3	**83**
Other cancers (46)	1	14	3	24						2				1		34	**79**
COPD (76)	1		2		2		3	3	2	3	54			2		5	**77**
Other digestive diseases (81)	3	1	2			2	1		2		1	16	17	1	1	27	**74**
Other respiratory diseases (77)	5		2	1	4	1	5		8	3	12	3		3	1	25	**73**
Other heart diseases (68)	1		1		1	4	15	14	4	1	4	1	1	5	1	18	**71**
Liver cancer (31)		58		2			1					3				4	**68**
Other infectious diseases (25)	18			1	3			1	5	1	1	1	1	3		17	**52**
Tuberculosis (5)	20				1						2					17	**40**
Other nervous system disorders (61)	10				2			1	4			1				10	**28**
Diabetes (52)				1	16		2		1	1				2	1	2	**26**
All other causes	14			8	9	8	5	3	18	1	1	9	4	6	2	294	**424**
																	
**Total**	**256**	**85**	**111**	**52**	**199**	**69**	**267**	**82**	**386**	**112**	**159**	**147**	**64**	**159**	**213**	**955**	**3316**

* Figures in column headings indicate ICD code for causes of death as per ICD Mortality Tabulation List 1 (see matched figures in parentheses in row headings, and reference 13 in manuscript)

† 66 = Hypertensive diseases

In addition to bias resulting from problems with generalizability, the inferences drawn from this study may well be affected by biases introduced during data collection or processing. One potential source of uncertainty lies in the quality of medical records and evidence that support the reference diagnoses used to infer the true underlying cause of death in the sample. Our findings revealed that across all causes in the sample, confirmatory evidence was available in 72% of cases. The diagnosis was based on weak evidence in only 20% of cases, although the observation that no opinion was possible for another 8% of cases suggests that the true extent of weak evidence may be somewhat greater than this. Further analysis identified that for all causes, sensitivity scores for registration diagnoses were higher for deaths with confirmed evidence, as would be expected. These findings indicate that while the available evidence is satisfactory for most leading causes of death, there is a clear need to improve the standard of medical records maintenance in hospitals in Thailand.

From another perspective, bias could result from inconsistency in the selection and coding of underlying causes of death in the study, particularly since this function was performed by study personnel at the provincial level (nine teams in all). Table 4 provides details of instances where the central team of coders identified and rectified inaccuracies in the application of selection rules and/or specific codes assigned to underlying causes of death by staff at the provincial level. Such changes were required more often in the provinces with smaller samples of deaths. Although about 17% of codes were changed overall, the change was only sufficient to alter cause categories at the WHO Mortality Tabulation List 1 level of aggregation (the aggregation level used for primary analyses in this study) in 3.1% of deaths. These findings support the broader national generalizability of this research, and at the same time justify the inclusion of provincial staff in implementing data processing activities, thereby enhancing national capacity for conducting future research of a similar nature.

**Table 4:** Changes in ICD codes for underlying causes of hospital deaths in study sample by province, based on review by central team of expert coders.

		Coding review		
		
Province	MR Review	Incorrect code ^a^ (%)	Disagreement ^b^ (%)	Changed code (%)
Ubolrajthani	606	11.1	1.8	12.9
Leoi	187	42.8	4.8	47.6
Chiang Rai	477	14.3	2.1	16.4
Payow	156	25.0	6.4	31.4
Supanburi	540	16.1	3.0	19.1
Nakhon Nayok	197	14.7	8.6	23.4
Songkhla	417	13.7	2.9	16.5
Chumpon	116	19.8	3.4	23.3
Bangkok	620	3.7	2.4	6.1
Total	3316	14.3	3.1	17.4

^a ^Incorrect - Change in ICD code at 3 or 4 character level, but no change at Mortality Tabulation List 1 level of aggregation

^b ^Disagreement - Change in ICD code resulting in change at Mortality Tabulation List 1 level of aggregation

Based on these observations about study design and implementation, we have estimated the probable age-, sex-, and cause-specific patterns of mortality among hospital deaths in Thailand as shown in Tables 5 and 6 for males and females, respectively. The figures in each cell in the tables represent the proportion of the age- and cause-specific deaths among all study deaths for males and females, respectively.

**Table 5:** Proportionate mortality by cause across broad age groups based on medical record review of hospital deaths in the study sample for males, Thailand, 2005.

		age group (years)					
		
Medically certified causes of death	ICD-10 codes	<1	1-14	15-49	50-74	≥75	All ages
Cerebrovascular diseases	I60-I69			2.0	5.5	3.1	10.6
Transport accidents	V01-V99		0.4	6.8	1.3	0.4	8.9
HIV/AIDS	B20-B24		0.2	7.3	0.6	0.0	8.1
Ischaemic heart diseases	I20-I25			0.8	4.2	1.9	6.9
COPD	J40-J47			0.5	3.3	2.6	6.4
Diseases of the liver	K70-K76			3.1	2.2	0.4	5.7
Cancer of lung	C33-C34			0.7	2.6	0.8	4.1
Pneumonia	J12-J18			0.8	1.7	1.1	3.6
Diabetes mellitus	E10-E14			0.4	2.3	0.8	3.5
Diseases of Genito Urinary system	N17-N98			0.5	1.7	1.1	3.2
Cancer of liver	C22			0.8	2.3	0.1	3.2
Other heart diseases	I26-I51		0.1	0.8	1.1	0.2	2.2
Assault	X85-Y09		0.1	1.7	0.2	0.0	1.9
Other Cancers	*			0.5	1.1	0.3	1.9
Hypertensive diseases	I10-I13			0.2	0.8	0.8	1.8
Other dis. of digestive system	K00-K22, K28-K66, K80-K92		0.1	0.4	0.7	0.7	1.8
Falls	W00-W19			0.7	0.7	0.3	1.8
All other external causes	**	0.1	0.1	0.8	0.6	0.2	1.7
Respiratory tuberculosis	A15-A16			0.4	0.6	0.5	1.5
Perinatal conditions	P00-P96	1.5		0.0	0.0	0.0	1.5
All others		0.7	0.9	6.0	8.3	4.1	20.0
**Total (%)**		**2.3**	**1.6**	**35.0**	**41.7**	**19.2**	**100.0**

**Total sample deaths**		**47**	**32**	**681**	**810**	**374**	**1944**

* Other Cancers: C37-C41, C44-C49, C51-C52, C57-C60, C62-C66, C68-C69, C73-C81, C88, C96-C97

** All other external causes: W20-W64, W75-W99, X10-X39, X50-X59, Y10-Y89

**Table 6:** Proportionate mortality by cause across broad age groups based on medical record review of hospital deaths in the study sample for females, Thailand, 2005.

		age group (years)					
		
Medically certified causes of death	ICD-10 codes	<1	1-14	15-49	50-74	≥75	All ages
Cerebrovascular diseases	I60-I69			1.2	5.7	6.4	13.3
Ischaemic heart diseases	I20-I25			0.2	5.5	4.0	9.7
Diabetes mellitus	E10-E14			0.8	5.6	3.0	9.5
HIV/AIDS	B20-B24			6.5	0.6	0.1	7.3
Diseases of GU system	N17-N98			0.7	2.5	2.1	5.3
Pneumonia	J12-J18		0.1	0.4	1.3	1.4	3.1
Other heart diseases	I26-I51			0.7	1.3	1.0	2.9
Transport accidents	V01-V99		0.4	1.6	0.7	0.2	2.9
Cancer of cervix uteri	C53			0.8	1.5	0.4	2.7
Diseases of the liver	K70-K76			0.9	1.6	0.2	2.7
COPD	J40-J47				1.3	1.3	2.5
Hypertensive diseases	I10-I13			0.1	1.3	1.0	2.4
Cancer of lung	C33-C34			0.2	1.5	0.7	2.3
Other dis. of digestive system	K00-K22, K28-K66, K80-K92				1.2	1.0	2.1
Dis of muscle & connective tissue	M00-M99			1.2	0.6	0.3	2.1
Cancer of colon	C18-C21			0.1	1.1	0.5	1.7
Cancer of liver	C22			0.2	1.0	0.5	1.7
Cancer of breast	C50			0.4	1.1	0.1	1.6
Perinatal conditions	P00-P96	1.4		0.0	0.0	0.0	1.4
Septicaemia	A40-A41			0.3	0.4	0.5	1.3
All others		0.7	1.1	5.6	9.2	4.9	21.5
**Total (%)**		**2.1**	**1.6**	**21.8**	**45.0**	**29.4**	**100.0**

**Total sample deaths**		**30**	**23**	**297**	**614**	**401**	**1365**

For both males and females, HIV/AIDS and transport accidents dominate the cause-specific mortality profiles during early adulthood (15-49 years). Among women at these ages, musculoskeletal diseases are the third leading cause of death, the majority of these cases being due to systemic lupus erythematosus, an autoimmune connective tissue disorder. Assault, falls, and other external causes are important causes of avoidable death among males at these ages.

Cardiovascular diseases, cancers, and chronic respiratory diseases are the leading causes of death at ages 50-74 years as well as among the elderly. In women, cancer of the cervix is the sixth leading cause of death at these ages and the ninth leading cause of death at all ages. The proportion of deaths from diabetes mellitus among females is nearly three times the proportion among males, with the majority of these deaths in both sexes occurring at ages 50-74. Diseases of the liver and of the genitourinary system (essentially renal disorders) are among the leading causes of death at all ages for both sexes, widening the focus of noncommunicable diseases in Thailand that require public health control measures.

## Discussion

In almost all developing countries where death registration is functional, even for only a fraction of the population, it would be reasonable to expect that the correct diagnosis of causes of death would be highest for those deaths that occur in hospitals compared with those that occur elsewhere. In principle at least, the clinical history of the deceased ought to be more reliably and comprehensively described in the medical records of patients who were hospitalized prior to death, and particularly for those who died in the hospital. We sought to investigate this premise in Thailand where over 140,000 deaths, or one-third of all registered deaths in the country, occur in hospitals. We set out to ascertain the true cause of death based on a careful review of all available clinical material for a representative national sample of 2.4% (3,316) of these deaths.

Our findings have significant implications for measuring and describing the burden of disease in Thailand. For example, the true proportion of hospital deaths due to stroke is probably 11% to 12% based on our methods, yet the vital registration system suggests it is closer to 8%. Similarly, ischemic heart disease is probably underdiagnosed by about one-third among hospital deaths, transport accidents by about 150%, and HIV/AIDS by about 300%. These errors need to be urgently redressed to improve the quality of information on causes of death from vital registration data in Thailand. More systematic training of physicians about the importance of maintaining complete, reliable, and coherent medical histories, and using these records to competently diagnose the cause of death according to international standards, is a clear priority for the Thai Ministry of Public Health.

The leading causes of death in hospitals represent a mix of communicable and noncommunicable diseases as well as a relatively high proportion of deaths from injuries in males (more than 16% of all male hospital deaths). While these findings are to be expected in a country well along the path of epidemiological transition, the rank order and magnitude of these individual conditions clearly inform priorities for public health as well as clinical intervention programs in Thailand. In particular, the finding that stroke causes more deaths than ischemic heart disease is consistent with the East Asian pattern for cardiovascular disease mortality [14]. This finding should be confirmed by further epidemiological research, which could inform allied research into the etiology, risk factors, and concomitant primary and secondary disease prevention strategies for cardiovascular diseases in Thailand [15,18]. The significant mortality in hospitals from several other noncommunicable diseases (diabetes, ischemic and hypertensive heart diseases, diseases of the liver and kidney), especially at ages that by any measure constitute premature mortality, indicates the need for improved clinical, therapeutic, and case management protocols for these conditions and/or their clinical complications. The implementation of such protocols, supported by adequate technical resources and trained manpower, could reduce in-hospital mortality from these causes through high-quality clinical care. Similar management protocols may well be required to prevent mortality in some cases from less grievous forms of injury, although this will require further research through an assessment of injury severity scores at admission.

While our observations about the representativeness of the sample and apparent methodological rigor of the data collection are reassuring, some caution is required when extrapolating our findings to estimate national in-hospital mortality in Thailand. Although our sample is representative by age, sex and cause, it was drawn from only nine of 76 provinces in Thailand, or at the district level, from 28 out of 795 districts (about 4%). Certain provincial or district-based epidemiological variations may not be adequately captured in our assessment of hospital-based mortality in Thailand. We have accepted the medical record review of cases in the sample to be an accurate representation of truth, although the evidence in some cases was only based on physician observations, rather than medical investigations. Further, the limited numbers of sample deaths at young ages makes it very difficult to derive meaningful estimates of cause-specific mortality at these ages, although the comparatively (compared to adults) low levels of under-5 mortality prevailing in Thailand reduce the need for a greater cause detail at these ages [19].

The observation that almost 30% of deaths that occur in hospitals are assigned diagnoses (ill-defined, septicemia) that are of little public health value suggests that there are serious limitations with the current system for certifying and coding causes of death in health establishments. Although there are undoubtedly social and legal issues that might discourage the certification of deaths from HIV/AIDS [20] or causes related to alcoholism, for example, these causes account for only a small fraction of all deaths certified with nonspecific causes. Findings from an audit of 2,232 original death certificates filed at registration for deaths included in our study shed further light on the likely mechanisms underlying poor-quality cause-of-death data. When compared with cause-of-death certificates derived from medical record review, 51% of original death certificates contained certification errors, principally:

1) listing of only a mode of death rather than a cause of death in 221 cases (9.9%).

2) incorrect sequencing of causes of death, despite the mention of the actual underlying cause of death in 221 cases (9.9%).

3) inconsistency with underlying cause of death as determined from medical record review in 572 cases (25.6%).

Other errors frequently observed were the use of abbreviations, ambiguous terms, or poor handwriting, all of which create problems in the accurate selection of underlying causes of death from original death certificates filed at registration. Detailed analyses of these findings are being included in a forthcoming manuscript. In summary, this death certificate audit improves upon a similar assessment reported by Choprapawon in 2003, which measured agreement between causes of death from registration, hospital death certificates, and diagnoses noted from medical records [21], but did not include detailed medical record review to derive accurate reference causes of death for validation, as was done in this study. Our findings suggest that systematic training of physicians about the importance of cause-of-death certification and its principles (including the avoidance of common errors) is an urgent requirement in Thailand and elsewhere. Such measures could significantly improve the accuracy of causes of death filed at registration for hospital deaths, given that our study identified that medical records in Thai hospitals (at least in three-quarters of all cases in our sample) are likely to contain adequate information to identify causes of death. Additional measures to improve medical records maintenance could further the fulfilment of this objective.

Improved medical documentation could also facilitate the ascertainment of causes for deaths where the deceased had attended a hospital during the terminal illness but was discharged prior to death. Adequate record linkage, or even some form of discharge record documenting the diagnosis, could help improve the accuracy of registered causes for these deaths. However, such instances are only a fraction of all deaths outside hospitals. For all other such deaths, verbal autopsy (VA) methods are the only option for cause-of-death ascertainment. The performance characteristics of verbal autopsy in Thailand were tested in a subset (2,558) of the hospital deaths reported here, using the medical record review diagnoses as the reference standards. The findings from this VA validation component, and their use in adjusting findings from the application of VA methods to estimate cause-specific mortality for deaths outside health facilities in Thailand, are reported elsewhere- [22].

Periodic studies to verify and validate registered causes of hospital deaths would greatly increase confidence in their utility for monitoring the impact of prevention or treatment interventions, and for the estimation of cause-specific mortality patterns in Thailand, using methods tested in other settings facing similar issues with quality of vital registration data [23]. A further major benefit of field research as in this study is the identification of critical impediments and poor practices that inhibit collection of higher quality cause-of-death data. Such evidence is essential to target reform strategies that, if implemented, will lead to urgently required improvements in the quality of data on causes of death in Thailand.

## Competing interests

The authors declare that they have no competing interests.

## Authors' contributions

JP led the field work. CR designed the study along with ADL and YP. WP completed the audit component. JP, CR, YP, WP, and NS undertook analysis. CR drafted the initial manuscript. All authors collaborated in developing the final manuscript and approved the final version.
